# Biomimetic Prussian Blue Sensor for Ultrasensitive Direct Detection of Myoglobin

**DOI:** 10.3390/polym17050630

**Published:** 2025-02-26

**Authors:** Jacinta Ricardo, Abel Duarte, Stefano Chiussi, Gabriela V. Martins, Felismina T. C. Moreira

**Affiliations:** 1 CIETI-LabRISE, ISEP, Polytechnic of Porto, R. Dr. António Bernardino de Almeida, 431, 4249-015 Porto, Portugal; 1220168@isep.ipp.pt (J.R.); ajd@isep.ipp.pt (A.D.); 2 CINTECX, Universidade de Vigo, 36310 Vigo, Spain; schiussi@uvigo.gal

**Keywords:** surface molecular imprinting, Prussian Blue nanocubes, screen-printed electrodes, cardiac biomarker, myoglobin, redox-free liquid

## Abstract

This research presents a novel, cost-effective, and scalable approach for the direct detection of myoglobin (Myo) in point-of-care (PoC) applications. In this strategy, redox-active Prussian Blue nanocubes (PBNCs) are applied to a disposable platinum screen-printed electrode (Pt-SPE). Subsequently, a biomimetic sensing layer is generated by electropolymerization of ortho-phenylenediamine (o-PD) in the presence of Myo, which forms molecularly imprinted polymer (MIP) sites by cyclic voltammetry (CV). The electropolymerization process takes place in a potential range of −0.2 V to +0.8 V, for five cycles at a scan rate of 50 mV/s, in a 10 mmol/L o-PD solution. After polymerization, the electrode is incubated in trypsin for 2 h to create Myo-specifically imprinted cavities. The structural and morphological properties of the biomimetic layer were analyzed by Raman spectroscopy, Fourier transform infrared spectroscopy (FTIR), and scanning electron microscopy (SEM). The direct detection of Myo was analyzed by differential pulse voltammetry (DPV). The results showed a linear response to Myo concentrations ranging from 1.0 ag/mL to 10 ng/mL, a limit of detection (LOD) of 0.76 ag/mL, and a R^2^ value of 0.9775. The absence of an external liquid redox probe simplifies the sensor design, improves portability, and reduces the complexity of the assay, making it more suitable for PoC.

## 1. Introduction

Acute myocardial infarction (AMI) leads to heart muscle necrosis, triggering the release of specific biomolecules such as myoglobin (Myo) into the blood and urine [1]. Myo is one of the first biomarkers to rise after a heart attack, offering a highly sensitive method to detect or rule out AMI within 1 to 5 h of symptom onset. Additionally, Myo can be detected between 4 and 50 h post-AMI [1,2]. Any protocol for the determination of Myo at the point-of-care (PoC) must provide a short turnaround time to detect these rapid biochemical changes, while being cost-effective for routine use. The analytical method should deliver reliable data for both normal and abnormal Myo levels without complex sample pre-treatment. Myo cut-off values range from 100 ng/mL to 200 ng/mL [3,4,5], with higher levels varying from 420 to 2000 ng/mL in serum [6] and 450 ng/mL in urine [7].

Various enzymatic and immunoassay-based methods for Myo determination have been described in the literature [8,9,10,11,12]. Early techniques for blood Myo detection focused on immunoreactions, including radioimmunoassay [13,14], enzyme-immunoassay [6], and chemiluminescent immunoassay [9]. Subsequent developments aimed to create PoC devices have used different transducer principles while maintaining the immunochemical basis [15,16]. Immunoassays offer unparalleled selectivity and specificity compared to chemical methods, but they often lack the stability and cost-effectiveness associated with chemical approaches. One successful alternative to immunoassays is the use of plastic antibodies [17,18,19,20]. These biomimetic materials are synthesized by growing a solid polymer structure around a target compound, creating imprinted sites that are complementary in size and charges to the imprinted molecule, binding the analyte similarly to natural antibodies [21,22,23,24,25,26]. This concept has been successfully applied in the development of many biosensors, including those for Myo detection [17,19,20,26,27].

Electrochemical biosensors offer a promising alternative to conventional biochemical analysis methods. These sensors are characterized by fast response times, portability, and minimal equipment requirements, enabling PoC analysis. Typically, electrochemical biosensors consist of a conductive substrate combined with a suitable bio-recognition element that interacts selectively with the target compound during sample incubation [28,29,30,31,32]. The resulting interaction can be monitored directly, if the target is electroactive, or indirectly using a redox probe to detect changes in electrical properties resulting from the interaction [33,34,35,36,37]. Since few compounds are electroactive, most interactions are monitored indirectly using external probes such as iron (III), ferric hexacyanoferrate (II), or ruthenium hexamine, applied to the electrodes after sample incubation [38].

The development of electrochemical biosensors that operate without redox mediators offers several advantages. These include simplicity of the system by eliminating the need for additional reagents and reduced overall complexity. These biosensors, which can be achieved by incorporating the redox probe directly into the conductive substrate or layer, exhibit greater resistance to external interference, enhanced stability, and improved accuracy and sensitivity, leading to lower operational costs. Additionally, these sensors perform reliably across a wide range of environmental conditions and promote more sustainable practices. Relevant research by the authors has explored such systems. In 2018, a novel mediator-free electrochemical sensor was developed with carbon nanotubes modified with copper oxide (CNT-CuO) printed onto a carbon surface. The resulting electroactive film served as a substrate for the assembly of the bio-recognition element for the detection of Alzheimer’s biomarkers at the PoC [39]. Later, in 2023, Ben Hassin et al. described a liquid, redox-free approach using a graphene oxide-based composite combined with Prussian Blue nanocubes (PBNCs) cast onto a screen-printed carbon electrode to detect tau protein, demonstrating strong analytical performance [33].

The main objective of this work was to develop a redox-free biosensing device with plastic antibodies assembled onto the surface of platinum screen-printed electrodes (Pt-SPE) for PoC applications. The proposed biosensor, modified through self-assembly and molecular imprinting techniques, was evaluated using various electrochemical techniques and applied to the analysis of biological samples for cardiovascular diagnosis.

## 2. Materials and Methods

### 2.1. Reagents and Solutions

All chemicals were of analytical grade and deionized water (conductivity < 0.1 µS/cm) was employed. Potassium hexacyanoferrate III (K_3_[Fe(CN)_6_]), potassium hexacyanoferrate II 3-hydrate (K_4_[Fe(CN)_6_]), and sodium hydrogen phosphate dihydrate (Na_2_HPO_4_) were obtained from Riedel-de Haën (São Paulo, Brasil). Polyethylenimine (PEI, MW 60 kDa) was obtained from TCI (Tokyo, Japan), iron III chloride 6-hydrate (FeCl_3_) from Scharlau (Barcelona, Spain), and myoglobin (Myo, MW 17 kDa), o-phenylenediamine (oPD), and trypsin from Sigma Aldrich (St. Louis, MO, USA). Potassium chloride (KCl) and fetal bovine serum were obtained from Merck (Darmstadt, Germany), phosphate buffered saline (PBS) tablets from VWR (Carnaxide, Portugal), and glucose, bovine serum albumin (BSA), and troponin from Fluka (Fisher Scientific, Porto Salvo, Portugal).

Stock solutions of 10 mg/mL Myo were prepared in PBS buffer (1.0 × 10^−2^ mol/L, pH 7.4). The selectivity study used Myo solutions at a concentration of 0.1 µg/mL, prepared in the same buffer. Solutions of interfering species, at variable concentrations, were also prepared in PBS buffer. For this purpose, solutions of glucose (2.8 mg/mL), BSA (6.0 mg/mL), and troponin (500 ng/mL) were prepared.

### 2.2. Apparatus

The electrochemical measurements were conducted with a potentiostat/galvanostat (Metrohm Autolab, PGSTAT302N, Utrecht, The Netherlands) with a FRA2 module and controlled by NOVA 2.1 software. Pt-SPEs (DRP-550AT) with working and counter electrodes made of platinum and the reference electrode, as well as the electrical contacts made of silver, were purchased from DropSens (Oviedo, Spain). The working electrode had a diameter of 4 mm, and the SPEs were placed in a DropSens switch box (Oviedo, Spain), which interfaced the Pt-SPEs with the potentiostat/galvanostat for electrochemical assays.

Scanning electron microscopic analysis (SEM) was performed using the high-resolution (Schottky) scanning electron microscope Quanta 400 FEG ESEM/EDAX Genesis X4M (FEI Europe B.V., Eindhoven, The Netherlands).

Fourier transform infrared spectroscopy (FTIR) measurements were obtained using a Nicolet 6700 FTIR (Thermo Fisher Scientific, Waltham, MA, USA) spectrometer, coupled to a diamond-based attenuated total reflectance (ATR) accessory. Raman spectroscopy measurements were made with a Horiba Jobin Yvon LabRam HR800 (Japan-France cooperation, Palaiseau, France) spectrometer using 488 nm excitation at normal incidence, 50× objective, and 1800 lines of grating. Laser power was kept below 0.20 mW to avoid damaging the samples and peak shift caused by Raman laser induced heating. Spectra were calibrated to the 2330 cm^−1^ position of N_2_ in air.

### 2.3. Synthesis of the PBNCs

The PBNCs were synthesized by a relatively simple method [40]. With constant stirring, 10 mL FeCl_3_ (5 mmol/L, pH 1.1), 1 mL PEI (3%), and 10 mL K_3_Fe(CN)_6_ (5 mmol/, pH 1.1) were mixed. The resulting mixture was heated to 95 °C and kept under reflux for 3 h. A change in the color of the mixture from yellow to dark blue was observed, proving the formation of the nanocomposite. The mixture was then washed in ultrapure water and centrifuged 3 times. Finally, a clean Pt-SPE was modified with the synthesized material by dropping 10 mg/mL of PBNCs 3 times onto the working electrodes and curing them at 60 °C for 30 min.

### 2.4. Electrochemical Measurements

Cyclic voltammetry (CV), differential pulse voltammetry (DPV), square wave voltammetry (SWV), and electrochemical impedance spectroscopy (EIS) measurements were conducted with 5.0 × 10^−3^ mol/L of K_3_[Fe(CN)_6_] and K_4_[Fe(CN)_6_] in PBS buffer, at pH 7.4. For CV assays, the potential was scanned from −0.3 V to +0.6 V, with a 50 mV/s ramp. In the SWV studies, potentials were changed using the same parameters, which correspond to a frequency of 20 Hz and a step height of 150 mV. For the DPV studies, potentials were changed from −0.3 V to +0.5 V, with a ramp of 0.125 V/s, corresponding to the afore mentioned frequency and step height. EIS assays were finally conducted with the same redox couple [Fe(CN)_6_]^3−/4−^ at an open circuit potential (OCP), using a sinusoidal potential perturbation with amplitude of 0.01 V (RMS) and 50 different frequencies, logarithmically distributed over a frequency range of 0.1 kHz to 100 kHz. The impedance data were fitted to a Randles equivalent circuit using the implemented NOVA 2.1 software.

### 2.5. Design of the Plastic Antibody on the Pt-SPE

**Figure 1:**
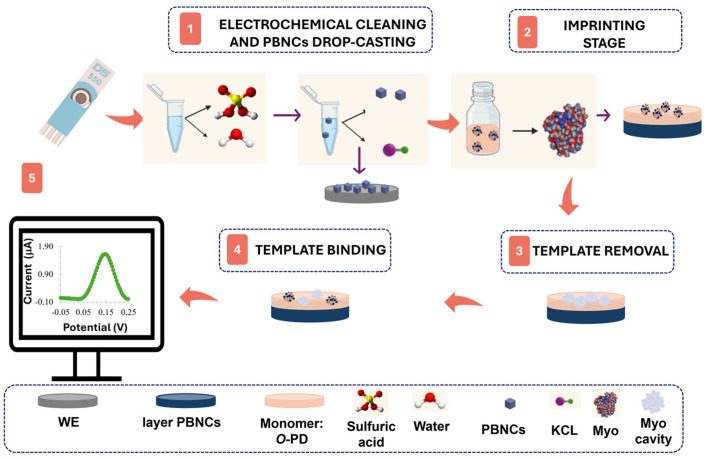
Synthesis of the molecularly imprinted polymer through its phases: (1) electrochemical cleaning and drop-casting of PBNCs, (2) imprinting stage via electropolymerization, (3) template removal, (4) template binding, and (5) electrochemical measurement.

Two different approaches were developed in this study. In the first approach, the platinum surface of the working electrode was cleaned and activated by sulfuric acid treatment via CV for 5 cycles, with a potential range from −0.1 V to +1.2 V (Figure 1, step 1). Then, three layers of PBNCs were cast onto the electrode surface and cured at 60 °C in an oven for 30 min (Figure 1, step 1). The imprinted material was obtained by electropolymerization (ELP) of a 10 mmol/L monomer solution in PBS on the PBNCs/Pt-SPE surface (Figure 1, step 2). The polymerization was carried out by CV over a potential range of −0.20 V to +0.90 V, at a scan rate of 50 mV/s, for 5 cycles.

Myo was removed from the polymeric matrix by incubation in a trypsin solution for 90 min at 37 °C (Figure 1, step 3). The film was then electrochemically cleaned with PBS buffer to remove protein fragments and adsorbed trypsin, followed by rinsing with deionized water. A negative control was also produced by excluding the protein adsorption step on Pt-SPE, resulting in non-imprinted polymers (NIPs).

The second approach followed the same protocol, but without the modification of the electrode with the PBNC layer.

### 2.6. Analytical Performance Evaluation

In this study, a novel concept was introduced in which conventional liquid redox probes are replaced by solid PBNCs on Pt-SPEs. The results obtained using both methods were compared to assess the benefits of incorporating PBNCs.

Calibration curves, with and without the liquid redox probe, were generated using SWV and DPV measurements, respectively, with Myo solutions ranging from 0.1 ag/mL to 10 µg/mL in PBS buffer (pH 7.4). A volume of 10 µL of each standard was incubated for 20 min on the working electrode of the Pt-SPE.

Calibration in 10,000-fold diluted fetal bovine serum was conducted using DPV measurements under the same conditions described earlier.

Selectivity studies were performed by a competitive assay between Myo at a concentration of 10 mg/mL and other interfering species.

## 3. Results and Discussion

### 3.1. Electrochemical Follow-Up of the Biosensor Assembling

**Figure 2:**
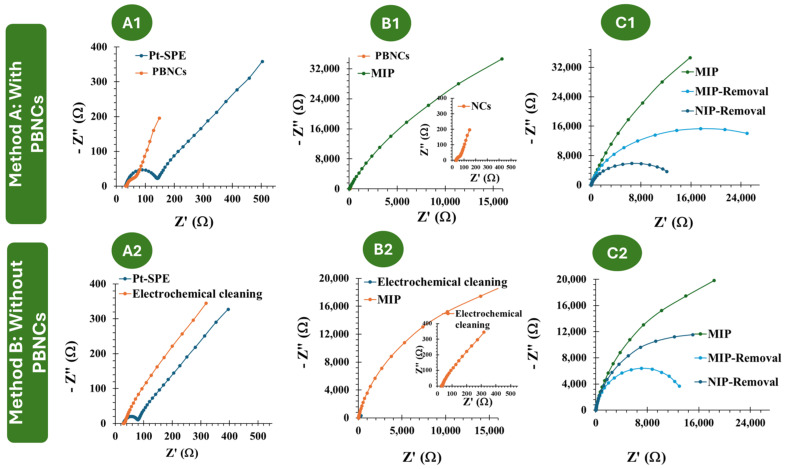
(**A1**–**C1**): EIS follow-up of the MIP and NIP assembly on the PBNC-based electrodes, in 5.0 × 10^−3^ mol/L K_3_[Fe(CN)_6_] and 5.0 × 10^−3^ mol/L K_4_[Fe(CN)_6_] in PBS buffer. (**A2**–**C2**): EIS follow-up of the MIP and NIP assembly on electrodes without PBNCs, in 5.0 × 10^−3^ mol/L and 5.0 × 10^−3^ mol/L K_4_[Fe(CN)_6_] in PBS buffer.

The immobilization of organic films on metal surfaces results in significant modifications of their electrical properties. These changes can be measured by observing the variations in the electron transfer capabilities of well-known redox systems, such as [Fe(CN)_6_]⁴^−^/[Fe(CN)_6_]^3−^ [41]. In this study, we introduced a novel concept where conventional liquid redox probes are replaced by solid PBNCs on Pt-SPEs (Figure 1) and compared the results obtained with both of them. EIS studies were employed to monitor the changes in Pt-SPE after each chemical modification (Figure 2).

The two approaches employed in this study, referred to as Methodology A and Methodology B, involved Pt-SPEs with and without PBNCs, respectively. In Methodology A, MIP and NIP materials were developed in the presence of PBNCs to understand the effect of these nanostructured materials on the electrochemical performance of the biosensor. According to Figure 2(A1–C1), casting the PBNCs onto the electrode surface led to a decrease in *Rct* (73.8 Ω ± 20%), indicating that the nanomaterial enhances electron transfer and reduces resistance. This demonstrates the good electrochemical properties of the PBNCs, as the *Rct* for Pt-SPE was 140.7 Ω ± 16.0 %. After the imprinting stage, during the polymerization of the monomer ortho-phenylenediamine in the presence of Myo, a significant increase in overall resistance was observed (1.27 × 10^5^ Ω ± 10 %), consistent with the insulating nature of the polymer, as reported in previous studies by the group [42,43]. After treatment with trypsin, a significant decrease in resistance was observed (1.63 × 10^4^ Ω ± 14%), which can be attributed to the absence of protein and the presence of imprinted cavities.

In Methodology B, i.e., without the PBNCs, the behavior is quite similar (Figure 2(A2–C2)). After MIP and NIP polymerization, the resistance increases significantly, as expected (62.4 × 10^4^ Ω ± 9.0% for MIP and 2.81 × 10^4^ Ω ± 11% for NIP). Upon template removal, there is a substantial difference in resistance of the MIP due to the absence of protein (3.38 × 10^4^ Ω ± 7%).

**Figure 3:**
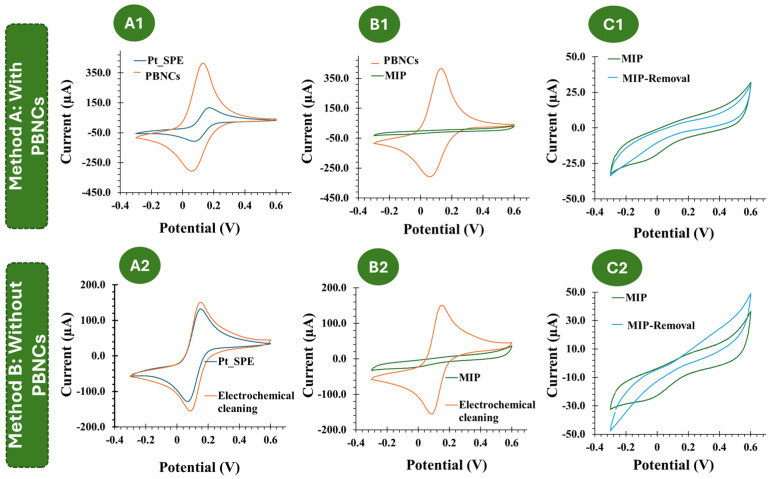
(**A1**–**C1**): CV follow-up of the MIP and NIP assembly on the PBNC-based electrodes, in 5.0 × 10^−3^ mol/L K_3_[Fe(CN)_6_] and 5.0 × 10^−3^ mol/L K_4_[Fe(CN)_6_] in PBS buffer. (**A2**–**C2**): CV follow-up of the MIP and NIP assembly on electrodes without PBNCs, in 5.0 × 10^−3^ mol/L K_3_[Fe(CN)_6_] and 5.0 × 10^−3^ mol/L K_4_[Fe(CN)_6_] in PBS buffer.

The CV assays, shown in Figure 3, support the previous EIS studies. Compared to the electrochemical behavior observed with the redox probe on the bare Pt electrode, the subsequent modification steps with the PBNCs exhibit a highly reversible process with very low peak separation (ΔV < 0.1 V), indicating rapid electron transfer. After the ELP process (Figure 3(B1)), no oxidation or reduction peaks were observed, which is consistent with the insulating nature of the polymer.

Considering each chemical modification applied to the Pt-based electrodes, without the inclusion of the PBNCs, a similar trend was observed (Figure 3(B2)). After MIP formation, no redox peaks were observed.

Overall, these results indicate that the PBNCs are highly electroactive and demonstrate a very high electron transfer rate, while the polymer shows very insulating behavior. Furthermore, both CV and EIS measurements indicated the formation of an insulating layer that behaves differently in the presence and absence of the nanomaterials.

### 3.2. Morphological and Chemical Characterization of the Biosensor Surfaces

#### 3.2.1. SEM

**Figure 4:**
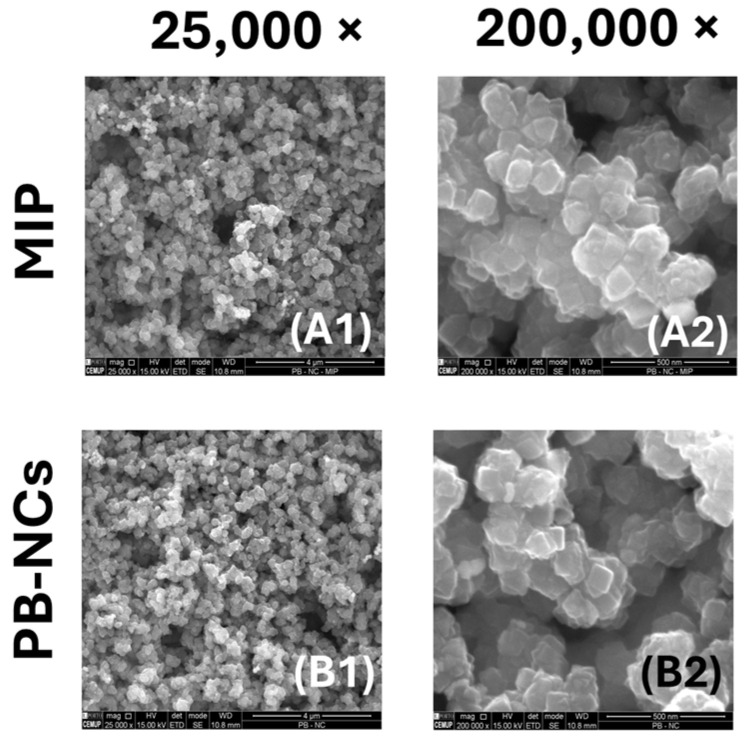
SEM analysis of the MIP and PBNCs materials. (**A1**) MIP amplified 25,000 times; (**A2**) MIP amplified 200,000 times; (**B1**) NIP amplified 25,000 times; (**B2**) NIP sensor amplified 200,000 times.

The SEM images were taken both for the nanomaterial PBNCs deposited on the Pt-SPE and for the MIP-sensitized modified electrode surface (Figure 4). Figure 4(B1,B2) clearly show the presence of well-defined nanocubes with an average size of 50 nm to 100 nm. Although the MIP-based polymer coating is not directly visible on the PBNC surface, Figure 4(A2) shows regions of increased brightness indicating the presence of the polymer. In addition, the nanocubes in Figure 4(A2) appear more aggregated and seem to be covered by a very thin polymer film, indicating successful MIP deposition.

#### 3.2.2. Chemical Characterization

**Figure 5:**
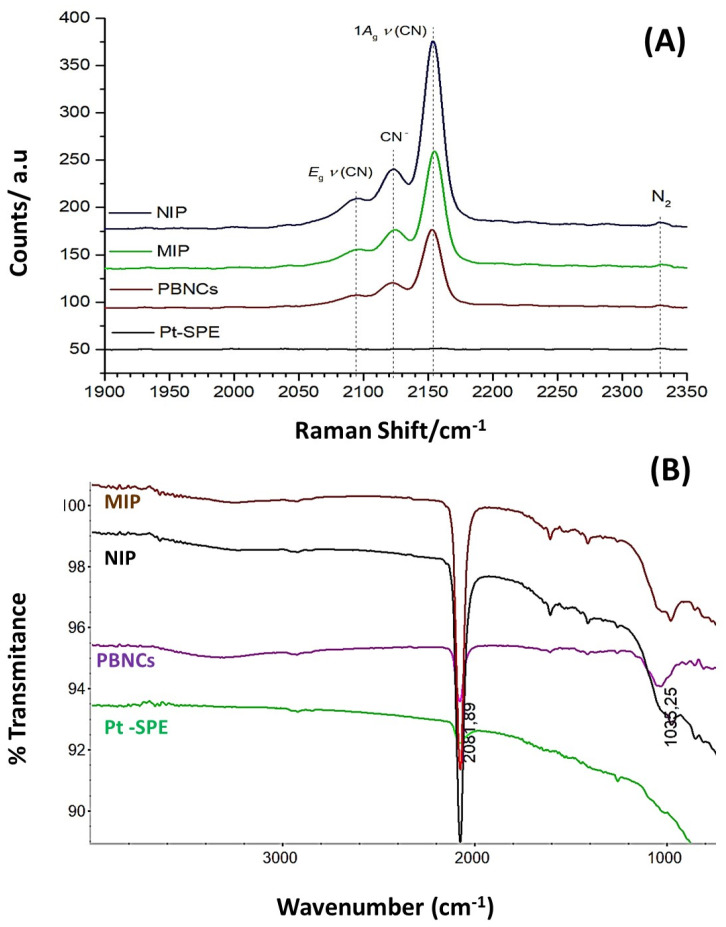
(**A**) RAMAN analysis, 488 nm Raman spectra, of the NIP, MIP, and PBNCs with Pt-SPE as background reference, taken at 0.20 mW laser power; and (**B**) FTIR analysis of the Pt-SPE, PBNC, MIP, and NIP materials.

Figure 5 displays the RAMAN and ATR-FTIR spectra for (i) Pt-SPE, (ii) Pt-SPE/PBNCs, (iii) Pt-SPE/PBNCs/MIP, and (iv) Pt-SPE/PBNCs/NIP.

The Raman spectrum (Figure 5A) for the bare electrode shows, as expected, no significant peaks, while the characteristic strong peak of the C≡N triple bond at around 2160 cm^−1^, as well as its typical Prussian Blue (PB) features [44] between 2070 cm^−1^ and 2200 cm^−1^, can be observed in all PBNC-treated Pt-SPE electrodes. These peaks are also present in the MIP and NIP sensors, as they are built on the Pt-SPE modified with PBNCs.

Prussian Blue (PB) is an iron (III) hexacyanoferrate (II) complex. Its characteristic blue color results from the intermittent charge transfer between iron (II) and iron (III) via the cyano groups [45,46]. The cyanide ligands in PB are coordinated with iron ions in different oxidation states (Fe(III)−CN−Fe(II)) with their specific vibrational stretching modes. Moretti et al. studied in detail the effects of Raman laser radiation on the associated Raman peaks and indicated that the oxidation state of the iron changes can be affected, leading to slight modifications in the peak position [44]. Raman spectroscopy is therefore a key technique used to characterize PBNCs, particularly through the presence of ν(CN) bands in the 2000 cm^−1^ to 2200 cm^−1^ range [47]. Consequently, the redox state of PB can be assessed by analyzing the Raman spectral characteristics of the ν(CN) peaks generated by the C≡N group that is coordinated to iron ions in different valence states. The primary peak, located between 2153 cm^−1^ and 2154 cm^−1^, corresponds to the 1Ag ν(CN) stretching vibration and the [Fe (II), Fe (III)] state. This peak shows a shoulder at lower wavenumbers (around 2122 cm^−1^), which is characteristic of the CN− group. A second peak observed at 2092 cm^−1^ to 2194 cm^−1^ corresponds to the Eg mode of the ν(CN) stretching vibration, which is also associated with the [Fe(II), Fe(III)] state [45,48]. The characteristic main low frequency peaks at 269 cm^−1^ and 533 cm^−1^ match fairly well with the ones mentioned by Moretti et al. and can be assigned to the Fe-CN-Fe deformation and the Fe-C stretching vibrations, respectively.

Because the cyanide ligands in PB are coordinated to iron ions of different oxidation states (Fe III−CN−Fe II), the wavenumber of the corresponding vibrational stretching modes can be related to the electronic structure of PB, and thus could also be used to evaluate electrochemical changes in the Pt-SPE sensors with PB.

Figure 5B shows the corresponding FTIR spectra, where a strong band around 2061.5 cm⁻^1^ can be observed in the PBNCs spectrum, which is associated with the C≡N stretching vibration, indicating the presence of cyanide groups [46]. The broad band centered at approximately 3250 cm⁻^1^ corresponds to the O–H stretching mode, which suggests the presence of interstitial water within the layers. Additionally, the band around 980 cm⁻^1^ observed in both the MIP and NIP sensors evidence C=C bending modes, confirming the presence of a polymeric matrix. However, it is not possible to distinguish between the MIP and NIP materials based on the FTIR spectra alone; the spectra only confirm the presence of the polymer.

### 3.3. Performance of the Biosensor in Buffer

The analytical performance of the biosensors developed for Myo detection, both in the absence and presence of the nanomaterial PBNCs, was evaluated by acquiring calibration curves. The SWV and DPV techniques were chosen over other voltametric methods due to their high sensitivity to electrode reactions, as well as their excellent detection capabilities and fast response time.

#### 3.3.1. Calibration Curve Without PBNCs

According to Figure S1 represented in the Supplementary Information, the current increases with the concentration of Myo. The expected behavior would actually be the opposite, as, considering the high molecular weight of the protein, one would expect an increase in resistance on the electrode surface due to the filling of the MIP cavities. This result can be explained by the fact that the isoelectric point of the protein (between 6.9 and 7.3) is very close to the pH of the PBS buffer (7.4) [49]. This causes the protein to have a charge close to neutrality, allowing some positively charged amino acids to be exposed and interact with the negative redox solution, resulting in an increase in current as the protein concentration increases. From the analysis of Figure S1B,C, we can conclude that the sensors start to show linearity with only 0.1 ag/mL; however, there are quite high standard deviations for the obtained values, with the errors for the NIPs being higher than those for the MIPs.

#### 3.3.2. Calibration Curve with PBNCs

**Figure 6:**
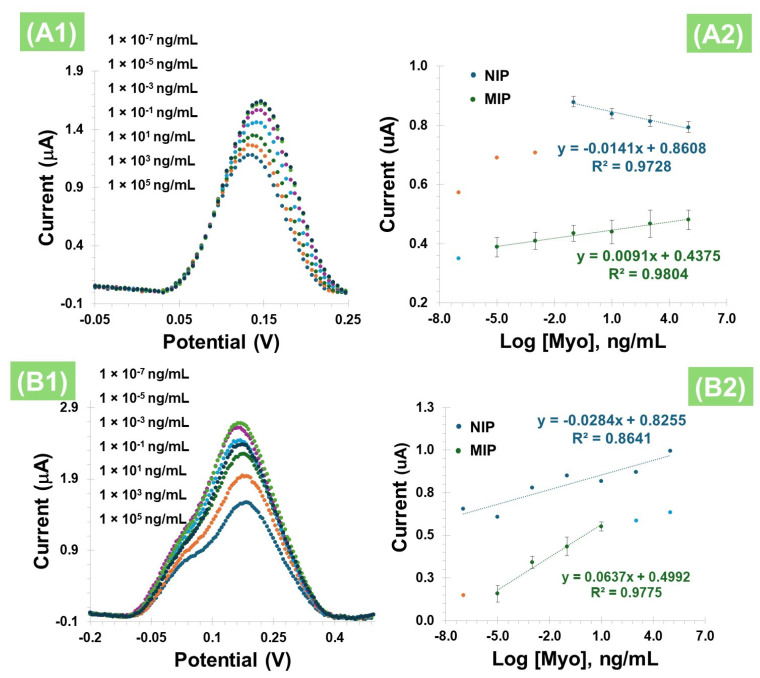
(**A1**) SWV measurement of MIPs in 5.0 × 10⁻^3^ mol/L K_3_[Fe(CN)_6_] and 5.0 × 10⁻^3^ mol/L K₄[Fe(CN)_6_] with different concentrations of Myo, and (**A2**) corresponding calibration curves obtained for NIP and MIP. (**B1**) DPV measurement of MIPs in PBS with different concentrations of Myo, without the liquid redox probe, and (**B2**) corresponding calibration curves obtained for MIP and NIP.

In terms of overall analytical performance, it was observed that the peak current increased with Myo concentration, a trend consistent across all tested concentrations (Figure 6(A1)), ranging from 1 ag/mL to 10 µg/mL, with a slope of 0.0091 µA/Dec and a limit of detection (LOD) of 0.63 ag/mL for calibration with liquid redox probe. The NIP showed the opposite behavior, with a slope of −0.0141 µA/Dec and a linear range between 0.1 ng/mL and 10 µg/mL (Figure 6(A2)). The relative standard deviation (RSD) was less than 12% for MIP-based and 6% for NIP-based sensors.

After evaluating the behavior of Myo with PBNCs on the electrode surface in the presence of a liquid redox probe, the equivalent study was conducted, but this time without this additional redox solution (Figure 6(B1)) and using the DPV technique. The presence of Myo in the imprinted polymer also resulted in an increase in the peak current (Figure 6(B2)) for both the MIP and the NIP. In terms of analytical performance, this effect was consistent across all tested concentrations, with a slope of 0.0637 µA/Dec, and a LOD of 0.76 ag/mL, evidencing a linear dependence between 1.0 ag/mL and 10 ng/mL, with an R^2^ value of 0.9775. These results suggest that the cavities were likely formed correctly and are complementary. In contrast, the NIPs exhibit randomness without forming a straight line (R^2^ value of 0.8641), which may be due to non-specific adsorption on the polymer surface.

A comparison of the results from Figure 6A,B, which show the electrochemical measurements taken in the presence and absence of the liquid redox probe, respectively, indicates that the liquid probe did not enhance the analytical response. In fact, the response slope is significantly lower compared to the direct measurements. This outcome may be attributed to interactions within the ferricyanide-based redox probe, which could potentially react with the PBNCs. Additionally, since Myo contains a porphyrin ring with an iron center, it is possible that this protein forms complexes with the ferricyanide or other iron species on the surface, thereby altering the electrochemical behavior. Therefore, for the subsequent assays, the methodology involving Pt-SPEs with PBNCs was established as the optimal condition for further studies.

#### 3.3.3. Selectivity Study

**Figure 7:**
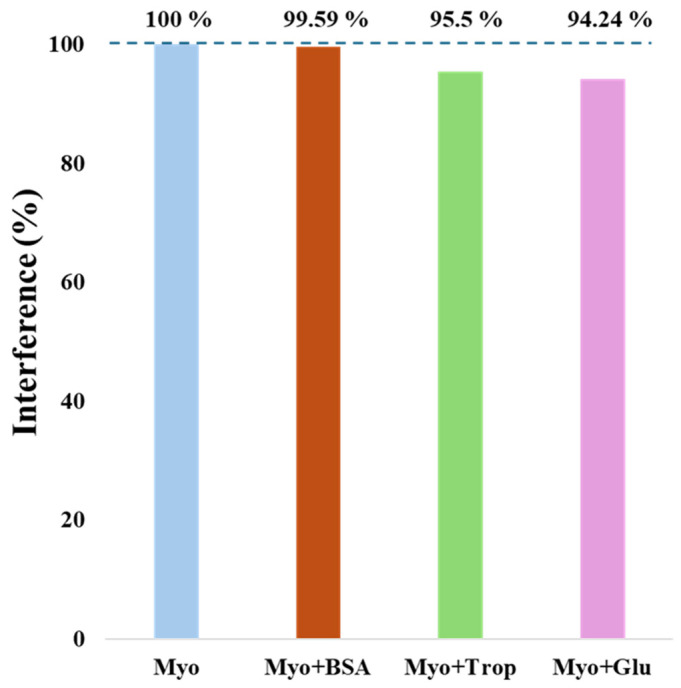
Representative graph of the selectivity test results for the interfering species: BSA, troponin, and glucose, in the presence of myoglobin.

For the evaluation of the biosensor’s suitability to distinguish Myo from other interfering molecules present in biological samples, selectivity tests were conducted. Competitive assays were performed comparing Myo at a concentration of 1.0 ng/mL with other interfering species (Figure 7). The selected interferents were BSA (6 mg/mL), glucose (2.8 mg/mL), and troponin (500 ng/mL). Figure 7 illustrates the selectivity study, where Myo alone or in a mixture was incubated on the biosensor surface for 20 min, showing only minor effects from the respective interferents. The greatest interference effect was observed with glucose (5.76%), followed by troponin (4.5%) and BSA (0.41%) as having the least interference. These results demonstrate that the optimized biosensor is selective for detecting Myo, even in the presence of the tested interferents, enabling its application for Myo detection in real biological samples.

### 3.4. Direct Calibration of the Biosensor in Serum

As a preliminary assay, calibration curves were generated in fetal bovine serum spiked with varying concentrations of Myo, without using the liquid redox probe. The serum was filtered and diluted 10,000 times. The results, detailed in the Supplementary Material as Figure S2, demonstrated that the presence of Myo in the imprinted polymer within the serum matrix led to an increase in the peak current. Moreover, the MIP biosensor showed a linear response between 0.1 ag/mL and 1.0 ng/mL, with an average slope of 0.0271 µA/Dec and an R^2^ value of 0.9976.

## 4. Conclusions

Cardiovascular diseases are still one of the most common causes of death worldwide, with heart attacks accounting for a significant proportion of the mortality rate. Early and accurate diagnosis of AMI is critical for enhancing patient outcomes, reducing mortality, and improving quality of life. While there are several diagnostic methods for cardiovascular disease, many are complex, costly, and impractical for routine use at the PoC. To overcome these limitations, an ultrasensitive direct electrochemical biosensor is presented that can detect Myo, one of the earliest cardiac biomarkers released during an AMI. In this study, a novel, cost-effective, and scalable sensor for the direct detection of Myo in PoC applications is presented. By integrating redox-active PBNCs with a MIP layer, the sensor achieves high specificity and sensitivity and detects Myo concentrations up to 1.0 ag/mL, with a wide dynamic range up to 10 ng/mL and an R^2^ value of 0.9775. Structural and morphological characterization confirmed the successful formation of the biomimetic layer, while DPV demonstrated the reliable performance of the sensor. A major advantage of this approach is the lack of an external liquid redox probe, which not only simplifies the design of the sensor, but also increases its portability and reduces the complexity of the assay. These features make the proposed sensor ideal for rapid on-site Myo-screening and pave the way for its potential application in the early diagnosis and monitoring of heart disease.

## Data Availability

Data is contained within the article or supplementary material.

## Supplementary Materials

The following supporting information can be downloaded at: https://www.mdpi.com/article/10.3390/polym17050630/s1, Figure S1: (A) SWV measurement of MIP in 5.0 × 10⁻^3^ mol/L K_3_[Fe(CN)_6_] and 5.0 × 10⁻^3^ mol/L K_4_[Fe(CN)_6_] with different concentrations of Myo in PBS. (B) Calibration curve corresponding to MIP and (C) calibration curve corresponding to NIP; Figure S2: (A) DPV measurement of MIPs incubated in fetal bovine serum spiked with different concentrations of Myo, measured in PBS, without the liquid redox probe. (B) Calibration curve corresponding to MIP and (C) calibration curve corresponding to NIP.
