# Supplementary material for: Biomimetic Prussian Blue Sensor for Ultrasensitive Direct Detection of Myoglobin
Source: Polymers (Basel). 2025 Feb 26;17(5):630. doi: 10.3390/polym17050630 (PMC11902790; doi:10.3390/polym17050630)
Supplement: Supplementary file 1 [file polymers-17-00630-s001.zip › polymers-3466713-supplementary.pdf]

Supplementary Information

Biomimetic Prussian Blue Sensor for Ultrasensitive Direct Detection of Myoglobin

Jacinta Ricardo[1], Abel Duarte[1], Stefano Chiussi[2], Gabriela V. Martins*[1] and Felismina T. C. Moreira*[1]

[1]*CIETI/LabRISE, School of Engineering, Polytechnic of Porto, R. Dr. António Bernardino de Almeida, 431, 4249-015 Porto, Portugal;*

[2]*CINTECX, Universidade de Vigo, Vigo, 36310, Spain;*

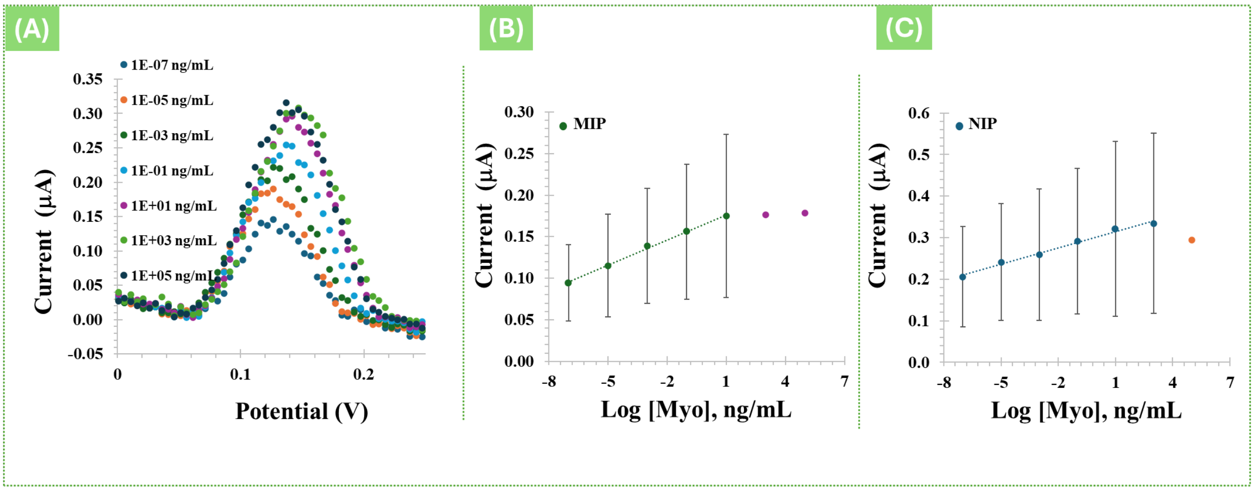

Figure S1. A) SWV measurement of MIP in 5.0 x 10^{-3} mol/L $K_3[Fe(CN)_6]$ and 5.0 x 10^{-3} mol/L $K_4[Fe(CN)_6]$ with different concentrations of Myo in PBS. B) Calibration curve corresponding to MIP and C) calibration curve corresponding to NIP.

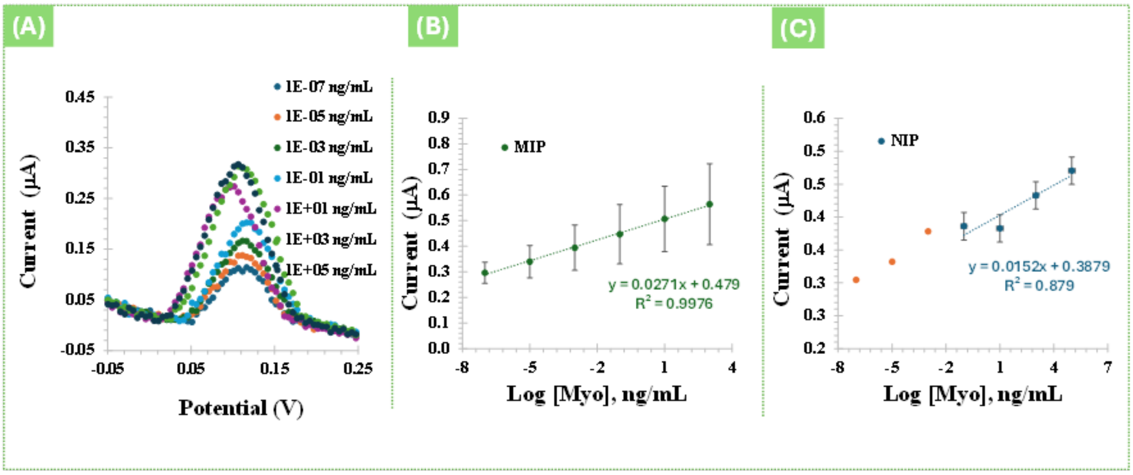

Figure S2. A) DPV measurement of MIPs incubated in fetal bovine serum spiked with different concentrations of Myo, measured in PBS, without the liquid redox probe. B) Calibration curve corresponding to MIP and C) calibration curve corresponding to NIP.
